# Activities of Serum Magnesium and Thyroid Hormones in Pre-, Peri-, and Post-menopausal Women

**DOI:** 10.7759/cureus.6554

**Published:** 2020-01-03

**Authors:** Bhagavan Reddy Kolanu, Sabitha Vadakedath, Venugopal Boddula, Venkataramana Kandi

**Affiliations:** 1 Biochemistry, Prathima Institute of Medical Sciences, Karimnagar, IND; 2 Clinical Microbiology, Prathima Institute of Medical Sciences, Karimnagar, IND

**Keywords:** magnesium, menopause, hormonal imbalance, thyroid hormones, tri-iodothyronine (t3), hypomagnesemia, tetra-iodothyronine (t4), post-menopausal women, thyroid stimulating hormone (tsh)

## Abstract

Introduction: Females go through a complex hormonal variation once they reach menarche. The menstrual cycle repeats every month regularly and is dependent on the normal functioning of the hypothalamus, pituitary, and ovarian hormones. The overall wellness of the females during the menstrual cycle depends greatly on nutritional status. It is common that women develop menstrual cycle-related symptoms and are routinely prone to thyroid dysfunction. The present study is carried out to assess the activities of Mg and thyroid hormones in pre-, peri-, and post-menopausal women.

Methods: A total of 165 women were recruited in the study after satisfying the inclusion criteria. An equal number of age-matched subjects were included as controls. All the subjects included in the study were selected from the patients attending various out-patient departments of the Prathima Institute of Medical Sciences, Karimnagar, Telangana, India. Blood samples from each subject were collected and analyzed by a semi-automated analyzer for the activities of Mg, and thyroid hormones tetra-iodothyronine (T_4_), tri-iodothyronine (T_3_), and thyroid-stimulating hormone (TSH).

Results: There was a statistically significant relationship between the serum Mg activities and the thyroid hormones between the study subjects and the control group. The activities of the serum Mg (1.72±0.33) in relation to the TSH (5.09±7.54) in the cases were found statistically significant (p <0.001) when compared to the serum Mg (1.8±0.20) in relation to the TSH in the control group (2.41±2.05). The activities of Mg were noted to fall in women through the peri (1.70±0.43), and postmenopausal age (1.60±0.34). There was a significant increase in the activities of TSH in women of premenopause (4.27±5.76), perimenopause (5.65±8.53), and postmenopausal age (7.19±11.07).

Conclusion: From the results of the present study, it can be concluded that the women reaching menopause could suffer from hypomagnesemia and inturn may develop thyroid and other hormonal disorders.

## Introduction

Women undergo several physiological and biological changes with the onset of menarche. They also experience mild to severe symptoms during the menstrual cycle which includes headache, myalgia, muscle cramps, intestinal irritability, sleep disturbances, and mood swings. Women have also been noted to suffer from thyroid dysfunction at various stages of their lives. The incidences of thyroid dysfunctions were also associated with age and menopause, as noted by increased incidences of thyroid dysfunction in post-menopausal women [1-2]. 

Thyroid hormones play an important role in regulating the basal metabolic rate (BMR) and calorigenesis. The calorigenic control by the thyroid gland is attributed to enhanced mitochondrial metabolism [3]. This causes stimulation of mitochondrial respiration and oxidative phosphorylation, thereby influencing the magnesium (Mg) to regulate the thyroid hormone functions. Mg is a mineral involved in energy-dependent reactions or ATP (adenosine triphosphate) generating reactions. Because of this, Mg is directly or indirectly involved in the regulation of more than 300 enzymatic reactions. The role of Mg in thyroid hormone synthesis could be indirect, i.e., it acts at the iodide uptake step and the deiodination step during the thyroid hormone synthesis [4].

Among the basic elements, the dietary iodine is involved in the synthesis of thyroid hormone. The dietary iodide is oxidized at or near the thyroid gland membrane into more reactive iodine, the free radical of iodine (l^-.^). Later the l^-.^ binds to the tyrosine molecules and then to thyroglobulin (thyroid protein) to form monoiodothyronine with the help of the thyroperoxidase enzyme. This process also involves sodium-iodide symporter, which in turn requires energy derived from the hydrolysis of ATP, thereby involving Mg, an essential cofactor for ATP generation [5].

Mg has also been noted to influence the deiodination process. During the deiodination process, the iodothyronine deiodinase and the iodotyrosine deiodinase enzymes require flavin mononucleotide (FMN) as a coenzyme and Mg to help the reduction process involving electron transport chain (ETC) [6-7].

The present study aims to assess the activities of serum Mg and thyroid hormones in pre-, peri-, and post-menopausal women.

## Materials and methods

A total of 165 female patients attending the outpatient departments (OPD) of Medicine, Surgery, and Obstetrics & Gynaecology were enrolled in the study. An equal number of age-matched subjects were recruited as the control group. The cases and the control subjects were categorized into three groups based on their age (≤29, 30-44, and >45 years). All the participants included in the study gave written consent, and the study was approved by the Institutional Ethical Committee (IEC).

The inclusion criteria for the cases included females with complaints of swelling around the neck region, menstrual irregularities, increased weight gain, and clinical thyroid disease. All the patients with hypertension, diabetes, and liver diseases and those who were pregnant were excluded from the study. The subjects with no clinical symptoms of thyroid disease, healthy women, and women with normal menstrual cycles were recruited as controls. 

Five milliliters of blood was collected from both the cases and the control subjects. The activities of serum Mg were estimated using a semi-automated analyzer (Photometer 5010) with DiaSys kits. A hormone analyzer supplied by Abbott (Abbott i1000SR Architect Plus) with chemiluminescence/magnetic particle immunoassay (CLIA) methodology was used to estimate the activities of thyroid hormones: tetra-iodothyronine (T_4_), tri-iodothyronine (T_3_), and thyroid-stimulating hormone (TSH).

Statistical analysis was performed using Microsoft Excel to derive the mean, standard deviation, and the *p*-value. 

## Results

The activities of the thyroid hormones among the cases (5.09 ± 7.54) and the control group (2.41 ± 2.05) were statistically significant (*p* < 0.001). The serum Mg activities among the cases (1.72 ± 0.33) and the control group (1.8 ± 0.2) were also found to be statistically significant (*p* < 0.001). The activities of the serum Mg and the thyroid hormones including the T_3_, T_4_, and TSH among the cases and the control group are shown in Table 1.

**Table 1:** The serum activities of Magnesium and the thyroid hormones among the cases and the control group TSH, thyroid-stimulating hormone; T_3_, tri-iodothyronine; T_4_: tetra-iodothyronine; Mg: magnesium; *: statistically significant

Parameter	Cases (n = 165)	Controls (n = 165)	p-value
Mg (mg/dL)	1.72±0.33	1.80±0.20	<0.001*
TSH (µIU/L)	5.09±7.54	2.41±2.05	<0.001*
T4 (µg/dL)	7.83±2.67	7.18±2.50	<0.001*
T3 (ng/mL)	1.35±0.94	1.54±1.12	<0.050*

The age-wise comparison of the parameters showed that the activities of Mg (1.60 ± 0.34) were comparatively lowered among post-menopausal women as compared to those under 29 years (1.68 ± 0.27). There was a significant rise in the activities of TSH in the post-menopausal women (7.19 ± 11.07) as compared to those who were under 29 years (4.27 ± 5.76). The serum activities of Mg among the post-menopausal women showed a significant difference (*p* < 0.0001) in the cases (1.64 ± 0.34) and the control group (1.80 ± 0.08). The activities of TSH (7.19 ± 11.07) in post-menopausal women were significantly higher (*p* = 0.0002) than those in the control group (1.50 ± 0.87) at the same age. The activities of Mg, TSH, T_3_, and T_4 _in various age groups are detailed in Table 2. 

**Table 2:** The activities of magnesium and thyroid hormones in different age groups of cases and the control group TSH, thyroid-stimulating hormone; T_3_, tri-iodothyronine; T_4_: tetra-iodothyronine; Mg, magnesium; *: statistically significant

Parameter	Age group (years)	Mean ± SD Cases (n=55 in each age group)	Mean ± SD Controls (n=55 in each age group)	p-value
Mg Normal Value: 1.5-2.3 mg/dL	≤29	1.68±0.27	1.77±0.22	=0.0580*
	30-44	1.70±0.43	1.80±0.17	=0.1117
	>45	1.60±0.34	1.80±0.08	<0.0001*
TSH Normal Value: 0.6-4.5 µIU/L	≤29	4.27±5.76	2.79±2.19	=0.0787
	30-44	5.65±8.53	1.14±0.97	=0.0002*
	>45	7.19±11.07	1.50±0.87	=0.0002*
T_4 _ Normal Value: 4.6-12.0 µg/dL	≤29	7.58±2.28	7.16±2.46	=0.5401
	30-44	7.87±1.84	7.27±2.98	=0.2152
	>45	8.67±3.72	8.40±2.20	=0.6481
T_3 _ Normal Value: 0.6-1.52 ng/mL	≤29	1.38±1.04	1.42±1.10	=0.8430
	30-44	1.13±0.35	2.40±1.14	<0.0001*
	>45	1.62±1.46	0.65±0.41	<0.0001*

## Discussion

Women experience various physiological changes once the menstrual cycle starts [8]. These changes are attributed to hormonal variations. During the menstrual cycle and after the menopause, most women complain of symptoms that range from mild to severe, which may include myalgia, headache, backache, abdominal cramps, bowel disturbances, and others [9]. Also, previous studies have observed that the women reaching menopause, those who have reached menopause, and older women could develop thyroid hormone dysfunction [1]. The mechanisms underlying the symptoms during the menstrual cycles, and the causes of thyroid dysfunctions in peri- and post-menopausal women have not been completely understood. 

In the current study, we have evaluated the serum Mg and thyroid hormones in the pre-, peri-, and post-menopausal women. The study had indicated that the serum Mg was found to decrease gradually with the age, as noted by low serum Mg activities among peri- and post-menopausal women. Also, the serum activities of Mg were low among the women in the case group as compared to the control group. The serum activities of Mg correlated well with the thyroid dysfunction, as noted by the decrease in the Mg, and an increase in the activities of TSH.

These observations justify the assumption that the serum Mg activities may be involved in causing the symptoms during the menstrual cycle and also be responsible for thyroid dysfunction in pre-, peri- and post-menopausal women. 

Probable role of Mg in causing the symptoms associated with the menstrual cycle and thyroid dysfunction

Magnesium is an abundant mineral found in the human body along with other essential elements like sodium, potassium, and the calcium which are available and absorbed through dietary sources. The dietary sources that are Mg-rich include nuts, grains, seeds, and leafy vegetables.

Mg is an essential macro element that functions as a cofactor for many enzymatic reactions [10]. It plays a critical role in mitochondrial oxidative phosphorylation and ATP synthesis. Animal experiments proved that the dietary supplementation of Mg significantly increases radioactive iodine uptake by thyroid cells; especially in subclinical hypothyroidism [11]. These findings emphasize that Mg deficiency may lead to decreased uptake of iodine by thyroid cells, thereby causing thyroid hormone disorders.

Mg helps in the balanced secretion of thyroid hormones and also plays a key role in the secretion of the active form of thyroid hormone T_3_. In a previous study which assessed the relation between Mg and thyroid function revealed that the serum Mg levels decrease in hyperthyroid state and increase during the hypothyroid condition [12]. An increase in the activities of Mg correlated with the thyroid hormone dysfunction, and the patients in such conditions were noted to excrete more Mg in the urine [12].

A previous report had suggested that during the transition from pre, peri, and post-menopausal states, there is a gradual estrogen dominance, which results in an imbalance between the ovarian hormones [13]. The raised estrogen levels in correlation with the progesterone activities cause estrogen dominance. Such a hormonal imbalance was previously attributed to reduced mineral supply, especially the Mg, Se (Selenium), and Iodine [14].

The role of Mg in estrogen dominance could be explained by its ability to clear estrogen from the liver in association with vitamin B6 (Pyridoxine). Mg was found to directly influence the conjugation of estrogen with glucuronic acid to enable its excretion into the bile and from the body. The major symptoms of menopause that include sleeplessness and mood swings were attributed to the role of Mg in tryptophan metabolism, i.e., the formation of serotonin and melatonin. Mg also helps in the balanced secretion of cortisol and progesterone [15].

The decline in the ovarian hormones (estrogen and progesterone) was linked to impaired gastrointestinal motility which in turn inhibits dietary absorption of Mg [16]. Mg is required in the synthesis of gamma-aminobutyric acid (GABA) which functions as a relaxant and helps in resolving stress and sleeplessness in women during and after menopause [17]. 

Mg modulates neurobiological mechanisms in the human body, including the neurotransmitter system and HPA (hypothalamus, pituitary, and adrenal gland) axis as evidenced from a previous study [18]. This HPA axis is a complex set of interactions between the hypothalamus, pituitary and the adrenal gland which controls the body’s response to stress and regulates the body processes like digestion, immunity, mood, emotions, and energy balance of the body [19].

It was also observed that exhaustion of minerals like zinc, Mg, B complex vitamins, and vitamin C in an adrenal gland may compromise the HPA axis [20-21]. As young females suffer from episodes of mood swings and sudden angriness which coincides with ovulation periods, the same symptoms are shown by the women during their pre-, peri-, and post-menopause age. Thus, it can be concluded that Mg plays a vital role in the regulation of hormonal activities including the thyroid hormones and the HPA axis.

**Figure 1:**
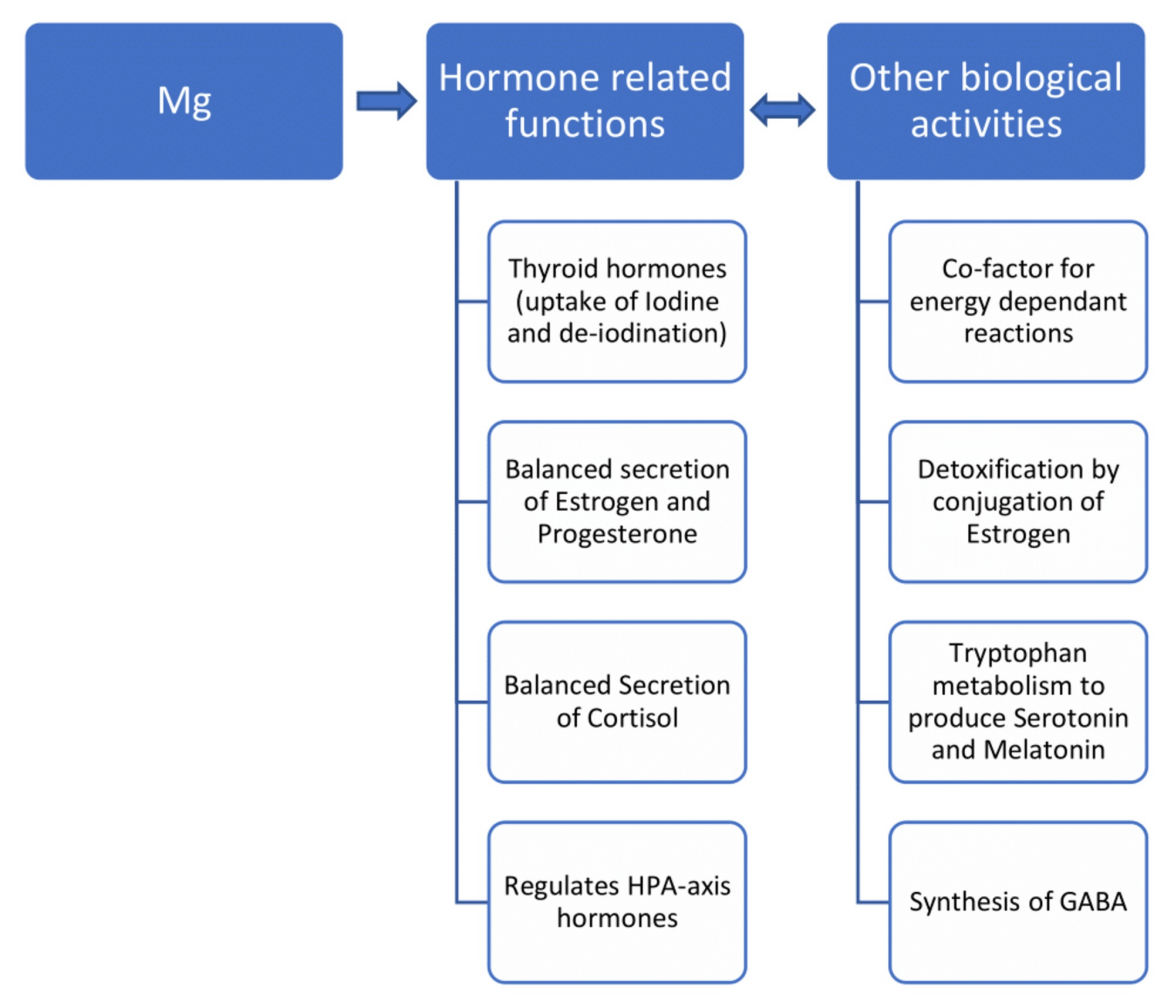
The schematic representation of the role of Mg in different human biological activities Mg, Magnesium; HPA-axis, hypothalamic pituitary adrenal-axis; GABA, gamma-aminobutyric acid

The activities of Mg may, therefore, appear significant in the normal functioning of various hormones that include the thyroid hormones, the sex hormones (estrogen, progesterone), the stress hormone/steroid hormone (cortisol), and the neurobiological hormones (HPA-axis hormones). Also, the Mg acts as a co-factor for various energy-dependent reactions, functions as a detoxicant by carrying out the conjugation of estrogen, participates in tryptophan metabolism that generates serotonin, and melatonin, and helps in the synthesis of GABA, the inhibitory neurotransmitter of the human brain. A schematic representation of the role of Mg in different human biological activities is shown in Figure 1. 

## Conclusions

This study demonstrated that there was a decrease in the activities of serum Mg in women through the peri- and post-menopausal age. Also, the activities of the TSH showed a significant increase in women through the pre-, peri-, and post-menopausal age confirming thyroid dysfunction. Activities of Mg showed a decline in the women with symptoms of the menstrual cycle as compared to healthy women in similar age groups. This study emphasizes that the serum Mg activities in pre-, peri-, and post-menopausal women could fluctuate and thereby influence the hormonal activities including the thyroid, sex, and other hormones. Regular evaluation of the activities of Mg and thyroid hormones through the pre-, peri- and post-menopausal age could be beneficial in the better management of the symptoms associated with the menstrual cycle. Further studies are required to improve the understanding of the role of Mg, the beneficial effects of nutritional/drug supplementation strategies of Mg, and its influence on the activities of various hormones.
